# Treatment Cessation in Chronic Myeloid Leukemia: Evidence and Uncertainties

**DOI:** 10.1002/hon.70155

**Published:** 2025-11-12

**Authors:** Jiří Mayer

**Affiliations:** ^1^ Department of Internal Medicine Hematology and Oncology University Hospital Brno and Masaryk University Brno Czech Republic

**Keywords:** chronic myeloid leukemia, treatment cessation, treatment‐free remission, withdrawal syndrome

## Abstract

Testing to discontinue imatinib already started some years after the advent of this new CML therapy. Since that time, despite many trials and studies in this field, there are still significant gaps, and many fundamental questions remain unanswered. Probably the most intriguing is the persistence of minimal residual disease, which does not lead to disease recurrence in all patients. Nevertheless, today's understanding enables TKI to be safely discontinued in eligible patients outside clinical trials. Notwithstanding, TKI cessation still has to be considered and indicated with caution, taking into account several important viewpoints, like: (i) why stop the therapy in a particular patient, and are all the eligible patients willing to cease the treatment? (ii) Will all the TKI‐related side effects relieve upon stopping? (iii) are there any side effects after discontinuing treatment? This review covers extensively all aspects of treatment cessation, like history, theoretical background, eligible patients, monitoring after treatment discontinuation, trigger for retreatment, therapy restart, predictive factors for successful therapy cessation, immunological aspects, potential complications of TKI withdrawal, late molecular relapses, blast crisis development, kinetics of preexisting TKI‐related side effects and laboratory values, and quality of life upon treatment cessation. The art of treatment cessation is to select the best candidate based on many diverse facts and information, and follow the patient in the most rational way with smartly anticipating the potential risks and side effects.

## 1 Introduction

In the past, chronic myeloid leukemia (CML) was considered an incurable disease with three typical phases: chronic phase (CP), accelerated phase and blast crisis, and with a short survival in advanced stages [1, 2]. Later, with the advent of allogeneic hematopoietic stem cell transplantation and interferon‐alfa, the prognosis improved. Interferon was able to induce a complete cytogenetic response in some patients who might become long‐term survivors [3]. However, the introduction of imatinib changed the treatment paradigm completely [4] and until now, tyrosine kinase inhibitors (TKI) are the mainstay of CML treatment.

With the accumulation of data in TKI‐treated CML patients it became apparent that a significant proportion of patients achieve long‐term deep disease suppression [5], and some of them may eventually stop treatment [6]; however, despite many trials and studies in this field, there are still significant gaps in our knowledge and many fundamental questions remain unanswered. The scope of this paper is to review the current data in detail regarding the first attempt at cessation in adult CML patients. If we consider stopping, we have to keep in mind several factors and aspects of this procedure. Actually, the first crucial question is why to stop the treatment at all? This can be due to some long‐term treatment side effects, the patient's wish, a planned pregnancy, or perhaps some financial issues from drug prices. But there are many other questions, like: (1) who is the best candidate for stopping treatment? (2) how can we predict the success of treatment discontinuation? (3) are all eligible patients willing to cease the treatment? (4) if we stop the treatment, how frequently and how long should patients be monitored? (5) will all the TKI‐related side effects relieve upon stopping? (6) are there any side effects after treatment discontinuation, like some new adverse events or disease progression? In the next text, these aspects of treatment cessation will be discussed based on current evidence with an emphasis on open questions.

## 2 Early Attempts of Treatment Discontinuation

Early attempts already started in the interferon era. Talpaz et al. [7] described 28 patients in complete cytogenetic response for at least 1 year of duration, of whom 18 patients continued to be in cytogenetic response after stopping treatment, while others experienced cytogenetic relapse. The reasons for treatment discontinuation were not specified. Mahon et al. [8] stopped interferon therapy in 15 patients after achieving complete cytogenetic response, of whom 8 relapsed. Interferon was ceased for side effects (*n* = 8) or patient's choice (*n* = 1), and in other patients for undetectable *BCR::ABL1* transcript. However, no information about the side effects' dynamic upon stopping was provided. Shortly after the introduction of imatinib there were reports on treatment discontinuation in a small series of patients with mixed results; the reasons for discontinuation were patients requests, side effects, or planned pregnancies [9, 10, 11]. In 2007, Rousselot et al. [12] published a study on 12 imatinib treated patients from 5 French centers which already had the features of a rigorous protocol: patients had to be in complete cytogenetic remission, and had an undetectable *BCR::ABL1* transcript for at least 2 years; after treatment discontinuation, patients were monitored monthly during the first 6 months and every 2 months thereafter. Molecular relapse was defined as *BCR::ABL1* positivity confirmed in 2 successive assessments. After treatment cessation, 6 patients (50%) relapsed during the first 6 months, and were re‐treated with imatinib. This was a clinical study exploring the feasibility of stopping imatinib. No information about the other reasons for treatment discontinuation or side effect kinetics upon stopping was provided.

These pioneering works paved the way to the current knowledge, and a flood of other publications followed. In fact, they all still have the imprint of the French work. Nevertheless, when interpreting the results of these studies, we have to keep in mind the possible differences in measuring the *BCR::ABL1* transcript, inclusion criteria, and also the trigger for re‐treatment.

## 3 Clinical Trials Testing the First Treatment Discontinuation Attempt

The collection of important trials is in the Tables 1 and 2 [13, 14, 15, 16, 17, 18, 19, 20, 21, 22, 23, 24, 25, 26, 27, 28, 29, 30, 31, 32, 33, 34]. The trials are sorted according to the date of publication and characterized by: trial name, number of patients, entry criteria, females/males ratio, age at study entry, duration of TKI therapy, duration of defined response, monitoring after treatment discontinuation, trigger for retreatment in the case of molecular relapse, treatment‐free remission (TFR) rate, prognostic factors, molecular response recovery after molecular relapse, and if detailed monitoring of TKI‐related side effects kinetics and/or of laboratory values was performed, and whether withdrawal syndrome frequency was reported. Despite there being a summary of 14 trials encompassing more than 2200 patients, clear, precise conclusions are rather surprisingly difficult to draw. The problem lies mainly in the different trials' designs, different populations of patients, and different follow‐ups. Nevertheless, some generalized points can be formulated: (1) treatment cessation is feasible, and the vast majority of trials stopped treatment abruptly; (2) the trials usually included patients in chronic phase without resistance to previous TKI therapy, and with no age limit; (3) the minimum TKI pre‐treatment was typically around 3 years; (4) achieving some defined treatment response was required, mainly MR4‐MR4.5; (5) as a trigger for re‐treatment, the loss of MMR (major molecular response) was generally selected; (6) after an abrupt discontinuation of treatment, the majority of molecular relapses occurred in the first 6–12 months, but late relapses did occur too; (7) the long‐term TFR rate is around 50%; (8) after stopping treatment, more frequent *BCR::ABL1* monitoring is employed; (9) among prognostic factors for successful TFR, the duration of previous TKI therapy and/or defined molecular response may play a role; (10) upon re‐treatment for molecular relapse, regaining the deep molecular response is typically quick; (11) the majority of trials did not monitor other aspects of finishing treatment except molecular recurrence.

**TABLE 1:** Collection of important clinical trials testing the first treatment discontinuation attempt. The trials are sorted according to the date of publication.

Author Publication year Trial name	Number of patients	Entry criteria	Trigger for retreatment	Remission	Monitoring after treatment discontinuation	Prognostic factors	Molecular response recovery after molecular relapse
Mahon et al., 2024 [13]; Saussele et al., 2018 [14]; EURO‐SKI	728	CML CP TKI > 3 years Confirmed DMR > 1 year Treatment switch for toxicity only	Loss of MMR	MRFS at 6 m: 61% MRFS at 36 m: 46%	Monthly first 6 m; Every 6 w until 12 m; Every 3 m for at least 3 years	For MRFS at 6 m: Duration of DMR (cutoff 3.1 y), Duration of treatment (cutoff 5.8 years), e14a2 transcript type; For MRFS at 36 m: Duration of DMR, Duration of treatment, Peripheral blasts at diagnosis, e14a2 transcript type	MR4 85% at 12 m for molecular relapse within 6 m; MR4 96% at 12 m for molecular relapse after 6 m
Shah et al., 2023 [15]; Shah et al., 2020 [16]; DASFREE	84	CML CP, dasatinib 1L or 2L ≥ 2 years, confirmed MR 4.5 ≥ 1 year, 1‐log reduction or < 10% of *BCR::ABL1* at 3 m of dasatinib^f^	Loss of MMR	TFR at 1, 2, and 5 years 48%, 46%, and 44%, respectively. No relapse after 39 m. Median (range) time to loss of MMR was 3.9 (1.1–38.9) m.^g^	Monthly for 12 m, and for every 3 m thereafter	Age ≥ 65 years, dasatinib 1L, dasatinib duration ≥ median	All evaluable patients regained MMR and MR4.5 in a median (range) of 1.9 months (0.9–3.7) and 3.3 months (1.5–29.6) respectively
Haddad et al., 2022 [17]; Single center retrospective study	284	CML CP Switch for resistance allowed. Entry criteria not exactly specified, but 281/284 pts were in MR 4.5 (54% of pts for ≥ 5 years)^c^	Loss of MMR	TFR 79% at 5 years	Monthly during the first 6 m, and then every 2 m for 6 m; or every 6–8 w during the first 6 m, and then every 3 m for 6 m	Deeper and longer molecular response; MR4.5 ≥ 5 years better TFR than MR4.5 < 5 years; MR4 ≥ 5 years better TFR than MR4 < 5 years;	Median time to regain undetectable transcript levels was 8 m
Hughes et al., 2021 [18]; Mahon et al., 2018 [19]; ENESTop	126^s^	CML CP; TKI ≥ 3 years, incl. imatinib > 4 w, nilotinib ≥ 2 years, no MR4.5 at time to switch to nilotinib, achieved MR4.5 on nilotinib; then 1 year of nilotinib consolidation; MR 4.5 at TFR study entry	Loss of MMR, confirmed loss of MR4	Of the patients who stopped nilotinib and entered the TFR phase, 57.9% were in TFR at 48 w. Of the 53 patients who had a loss of response and exited the TFR phase, 49 did so within the first 24 w. TFR at 5 years 42.9%.	Every m for the first y, every 6 w for the 2^nd^ y, and every 3 m thereafter	Multivariate logistic regression analysis of patient characteristics confirmed time since first MR4.5 until TFR entry as a statistically significant predictor of successful TFR at 48 w (odds ratio 1.033 [95% CI: 1.004, 1.063] for every month increase). Age < 56 years.	The majority of patients who reinitiated treatment rapidly regained MR4 and MR4.5. The median time to regain MR4.5 was 2.9 months (range, 0.9–22.5).
Radich 2021 [20]; Hochhaus 2017 [21]; ENESTfreedom	190	CML CP, frontline nilotinib ≥ 2 years, MR4.5, then 1 year of nilotinib consolidation; sustained deep molecular remission^t^	Loss of MMR	At the 5‐year cut‐off, 79 out of the 190 patients (41.6%) who entered the TFR phase remained in MMR or better without nilotinib treatment, with 76 (40.0%) remaining in MR4.5^u^	Every 4 w during the first 48 w, every 6 w during the next 48 w, and every 12 w thereafter	Patients with low Sokal risk score at diagnosis had a TFR rate of 50.8% at 5 years compared with 38.0% for patients with intermediate‐risk score and 27.6% for patients with high‐risk score. Longer history of nilotinib exposure and longer MR4.5 response was also associated with higher TFR rates. The TFR rate at 5 years was higher for patients who had remained in MR4.5 at w 48 of the TFR phase than for patients who had not (86.0% for patients in MR4.5 vs. 33.3% for patients in MR4 and 20.0% for patients in MMR at week 48 of the TFR phase). The response stability was also important for TFR rates at 5 years. Patients with stable MR4.5 during the first 48 w of the TFR phase had a TFR rate of 86.7%, compared with 36.4% for patients with at least one RQ‐PCR value showing loss of MR4.5 during the first 48 w of TFR.	Of the 91 patients who entered the treatment reinitiation phase, 90 (98.9%) regained MMR, most of them (91.2%) within the first 12 w of restarting nilotinib. Of those 91 patients, 84 (92.3%) regained MR4.5
Kimura et al., 2020 [22]; First line DADI	58	CML CP, sustained MR ≤ 0.0069%^d^ for 1 year on dasatinib first line, at least 2 years of dasatinib, then 1 year of dasatinib consolidation	Confirmed loss of MR 0.0069%^d^, or single loss of MMR	TFR at 6 m, as well as at 12 m, 55.2%	Monthly for 12 m; every 3 m for further 12 months	Lower CD4+ cell count for better TFR	All patients who received dasatinib rapidly achieved major molecular response and 23 (92%) of the 25 patients achieved deep molecular response, again within 12 months (median time 3.2 months)
Clark et al., 2019 [23]; Clark et al., 2017 [24]; DESTINY^j^	174	CML CP; received nilotinib, imatinib (85%) or dasatinib for ≥ 3 years; treatment switch for toxicity only; at least MMR for 3 measurements in the last 12 m	Confirmed loss of MMR	At 36 m, MRFS for MR4 group 72%, and for MMR group 36%^k^	Monthly for the first 2 years and in alternate months thereafter	MR4 subgroup; time on TKI^l^	Of the 64 recurrences across the trial, 91% returned to MMR within 5 months of resumption of TKI therapy, with no significant difference (*p* = 0.59) between the MR4 and MMR groups
Okada et al., 2018 [25]; Imagawa et al., 2015 [26]; DADI	63	CML CP, imatinib pretreatment, dasatinib as second+ line (for imatinib resistance or intolerance), no ACAs at dg, no dasatinib resistant mutations, stable MR < 0.0069% ≥ 1 year on dasatinib^d^	MR ≥ 0.0069%	TFR at 6, 12, and 36 m: 49%, 48%, and 44%, respectively	Monthly for 12 m; every 3 m for further 12 months; Then every 6 months	Absence of imatinib resistance^e^; greater number of cytolytic NK cells (CD16^+^CD56^+^)	All patients who restarted dasatinib or nilotinib demonstrated a rapid molecular response: 30 (88%) returned to MR4.0 within 3 months and 4 had regained MR4.0 by 6 months.
Etienne et al., 2017 [27]; Mahon et al., 2010 [28]; STIM1	100	CML CP imatinib > 3 years UMRD > 2 y^a^	Positivity of *BCR::ABL1* confirmed by a second analysis point that indicated an increase of one log in relation to the first analysis point at two successive assessments, or loss of MMR.	MRFS at 6 m: 43% MRFS at 60 m: 38%	Monthly first y; Every 2 m in the second y; Every 3 m thereafter	Sokal risk score (low + intermediate vs. high) Imatinib duration > 54 m	55/57 pts achieved UMRD in a median time 4.3 m (1.5–21) upon restarting therapy
Rea et al., 2017 [29]; STOP 2G‐TKI study	60^i^	CML CP, dasatinib or nilotinib 1L or 2L for imatinib intolerance, suboptimal response or resistance, ≥ 3 years of therapy, ≥ 2 years of uMR4.5^h^. Multicenter observational study	Loss od MMR	TFR rates at 12 and 48 m were 63.33% and 53.57%, respectively; median time to molecular relapse 4 m	Monthly during the first 12 m, every 2–3 m during the second y, and every 3–6 m for up to 5 years	TFR rates at 48 months were 79.78% and 18.18% for patients maintaining a MR4.5 at 3 months or not, respectively. Significantly worse TFR for those who receive the 2L therapy for prior suboptimal response or resistance	MMR, MR4.5, and uMR4.5 were regained after a median time of 2 m, 3 m, and 4 m, respectively
Lee et al., 2016 [30]; Park et al., 2016 [31]; Lee et al., 2013 [32]; KID study	Lee et al., 2013; *n* = 48; previous HSCT, *n* = 20^p^	CML CP, imatinib > 3 years, undetectable *BCR::ABL1* ≥ 2 y^o^, imatinib for relapse after allogeneic HSCT allowed	Molecular relapse, confirmed loss of UMRD^o^ or MMR	12 m probability of sustained MMR: 64.4% for non HSCT pts, and 100% for HSCT pts	Every m for the first 6 m, every 2 m up to 12 m, and every 3 m thereafter	HSCT; UMRD^o^ duration; imatinib therapy duration	All nine patients who lost MMR were retreated. Eight of these patients reestablished MMR at a median of 1.7 months after resuming therapy and seven of these patients reestablished UMRD at a median of 5.6 months.
	Lee et al., 2016; *n* = 90^q^	CML CP, imatinib > 3 years, undetectable *BCR::ABL1* ≥ 2 y^o^	Confirmed loss of UMRD^o^ or MMR; imatinib reintroduced if loss of MMR	The overall 12‐m and 24‐m probability of sustained MMR was 62.2% and 58.5%, respectively. The overall 12‐m and 24‐m probability of sustained UMRD was 50.0% and 50.0%, respectively. Among the 37 patients with molecular relapse, the median time to MMR loss was 3.3 m^r^	Every m for the first 6 m, every 2 m up to 12 m, and every 3 m thereafter	Presence of withdrawal syndrome, negativity of digital PCR at screening, and longer duration of imatinib therapy (< 62 m vs. ≥ 62 m)	All patients reestablished MMR at a median of 3.9 m after resuming therapy, and 32 reestablished UMRD at a median of 7.2 m
Ross et al., 2013 [33]; TWISTER	40	CML CP imatinib > 3 years UMRD > 2 y^b^	Loss of MMR, or two consecutive positive samples at any value	TFR 47.1% at 24 m; 68% of relapses in the first 6 m, 32% of relapses between 6 and 27 m	Monthly for 12 m; every second m for 12 months; Then every 3 months	Interferon pretreatment	All of the 22 patients who relapsed had at least one UMRD sample after restarting IM. The median time to the first UMRD sample was 3 m (range, 0–17 m)
Takahashi et al., 2012 [34]; multicentre retrospective study	43 out of 3242 imatinib treated patients^m^	Pts who stopped imatinib and were ≥ 6 m without molecular recurrence; CML CP with CMR^n^	Molecular recurrence, not specified	MRFS at 5 years 47%	Not specified	Duration of therapy (median 26.3 vs. 51.7 m); total imatinib dose; prior interferon; CMR duration (median 6 vs. 32.5 m)	TKI treatment was restarted in 17/19 pts with molecular recurrence who all then recovered to CMR (13 patients) or MMR (4 patients). The remaining 2 patients had shown sustained MMR with no therapy.

Abbreviations: ACAs = additional chromosomal abnormalities; CP = chronic phase; dg = diagnosis; DADI = dasatinib discontinuation trial; DESTINY = The De‐Escalation and Stopping Treatment with Imatinib, Nilotinib, or sprYcel study; ENEST = Evaluating Nilotinib Efficacy and Safety in Clinical Trials; EURO‐SKI = European Stop Kinase Inhibitors study; DMR = deep molecular response: *BCR::ABL1* transcript ≤ 0.01% on International Scale (IS); HSCT = hematopoietic stem cell transplantation, allogeneic; KID study = Korean Imatinib Discontinuation Study; L = line; m = month; MMR = major molecular response: *BCR::ABL1* transcript ≤ 0.1% on International Scale (IS); MR = molecular response; MRFS molecular recurrence‐free survival; pts = patients; STIM = Stop Imatinib Study; TFR = treatment‐free remission; UMRD = undetectable molecular (minimal) residual disease; w = week; y = year.

^a^ reported method sensitivity up to 10^−5^.

^b^ calculated detection limit 0.0035% (4.5 log).

^c^ elective discontinuation 70%, adverse events 24%, pregnancy 3%, diagnosis of second cancer 2%, other 1%.

^d^ MR < 0.0069% = MR 4.2.

^e^ TFR at 36 months was only 8% in imatinib‐resistant patients; it was 55.6% and 50.0% in imatinib‐intolerant patients and patients who had decided to switch to dasatinib, respectively. The median *BCR::ABL1* doubling time in imatinib‐resistant patients was significantly shorter than that in non‐resistant patients (16.3 days, vs. 52.3 days).

^f^ 44% of pts received dasatinib 1L, 56% of pts dasatinib 2L+: 53% for resistance, 38% due to intolerance.

^g^ No difference in TFR in dasatinib 2L switched for resistance or intolerance.

^h^ uMR4.5 = undetectable molecular transcript with method sensitivity of MR4.5.

^i^ intolerance to imatinib 65% of patients, suboptimal response or resistance 21.7% of patients.

^j^ The trial design comprised initial de‐escalation to half the standard TKI dose for 12 months, followed by complete cessation.

^k^ If all PCR measurements during this pre‐trial observation period were 0.01% or less (i.e., in MR4 throughout), the patient was assigned to the MR4 group; if some or all PCR measurements were greater than 0.01% but 0.1% or less, the patient was assigned to the MMR group.

^l^ The model estimated a non‐linear average decrease of around 8% in the proportion of recurrences per additional year on TKI in the trial overall, but this proportion differed according to trial group, with an average 4% decreased recurrence rate per year for the MR4 group and 11% for the MMR group.

^m^ The reasons for which imatinib was discontinued were adverse events (*n* = 18), patient's request due to cost (*n* = 14), patient's desire to become pregnant (*n* = 3), and long undetectable residual disease (*n* = 8).

^n^ CMR = complete molecular response, not detectable *BCR::ABL1* transcript with a method with a sensitivity < 0.01%.

^o^ UMRD = undetectable molecular residual disease with method sensitivity of at least MR4.5.

^p^ Imatinib started for hematological, cytogenetic, or molecular relapse after HSCT. Four pts received donor lymphocyte infusion, too.

^q^ Reason for IM discontinuation, (%): low‐grade adverse events (50), inconvenience of daily doses (3), medication cost (1), pregnancy/planning pregnancy (3), patient preference (42).

^r^ Eight patients who lost UMRD but not MMR exhibited different patterns of *BCR‐ABL1* kinetics: 7 patients spontaneously reachieved UMRD after a median time of 1.2 months (range, 0.7–5.4 months) and 1 patient showed *BCR‐ABL1* transcript fluctuation under the level of 0.1% for 20.6 months.

^s^ Reasons for switching from imatinib to nilotinib: intolerance 40%, resistance 24%, physician preference 36%.

^t^ defined as MR4.5 in the most recent assessment, ≤ 2 assessments between MR4 and MR4.5, and no assessment worse than MR4 in the last 4 quarterly RQ‐PCR assessments during the consolidation phase.

^u^ Out of 94 patients, 88 lost MMR during the first 48 weeks, with three additional patients experiencing loss of MMR in the second and third 48 weeks after discontinuing treatment. Four of the six patients who lost MMR after 48 weeks in TFR had lost MR4.5 during the first 48 weeks, while another had unstable MR4.5 during the same period; the remaining patient had stable MR4.5 at 48 weeks.

**TABLE 2:** Collection of important clinical trials testing the first treatment discontinuation attempt. The trials are sorted according to the date of publication.

Author Publication year Trial name	Number of patients	Females/males	Age at study entry, y	Duration of TKI therapy	Duration of defined response	Detailed monitoring of TKI‐related side effects kinetics	Detailed monitoring of laboratory values	Withdrawal syndrome frequency
Mahon et al., 2024; Saussele et al., 2018; EURO‐SKI	728	47/53%	60 (median) (19–89)	7.5 years (median) (3–14.1)	MR4 4.7 years (median) (1–13.3)	No	No	31% (35.6%)^a^
Shah et al., 2023; Shah et al., 2020; DASFREE	84	44/56%	52 (median) (24–80)	Any TKI 65 m (28–221) who lost MMR, 73 m (28–154) no loss of MMR; dasatinib 43 m (26–149) who lost MMR, 56 m (28–125) no loss of MMR; median (range)	Time in MR4.5 28.9 m (median) (13.0–243.7)	No	No	69%/11%^d^
Haddad et al., 2022 Single center retrospective study	284	51/49%	63 (median) (54–72)	117 m (median) (79–149)	MR4 74 m (median) (48–104); MR4.5 64 m (median) (39–91)	No	No	Not reported
Hughes et al., 2021; Mahon et al., 2018; ENESTop	126	56/44%	56 (median) (21–86)	87.7 m (median) (49–171)	Median time from achievement of MR4.5 with nilotinib to study entry 19.8 m (0.3–78)	Yes	Yes	Musculoskeletal pain‐related AE 10.5% at study entry versus 52.6% during the first y of TFR
Radich 2021; Hochhaus 2017; ENESTfreedom	190	49.5/50.5%	55 (median) 21–86	43.5 m (median) (32.9–88.7)	Time from achievement of MR4.5 with nilotinib to study entry, 18.3 m (median) (0.3–70.9)	Yes	Yes	Musculoskeletal pain increased in frequency from 16.3% of patients during the consolidation phase to 41.9% of patients in the first 48 w of TFR
Kimura et al., 2020; First line DADI	58	52/48%	Not reported	40.4 m (median) (38.1–51.1)	MR ≤ 0.0069% 23.3 m (median) (15.7–29.4)^c^	No	No	Not reported
Clark et al., 2019; Clark et al., 2017; DESTINY	174	44/56%	59 (median) (45–68)	6.9 years (median) 4.8–10.2	*BCR::ABL1*% at trial entry: 0.001 (median) 0.0006–0.003	Yes	No	40%
Okada et al., 2018; Imagawa et al., 2015; DADI	63	35/65%	59 (median) (24–84)	Duration of imatinib + dasatinib 82 m (median) (23–142)	Not reported	No	No	Not reported
Etienne et al., 2017; Mahon et al., 2010; STIM1	100	52/48%	59.4 (median) (29–81)	72.1 m (= 6 years) (median) (36–243)	UMRD 36.4 m (median) (24–107)	No	No	Not reported
Rea et al., 2017; STOP 2G‐TKI study	60	63.3/36.7%	60 (median) (26–81)	The median duration of TKI treatment was 76 m (36–153); the median duration of 2G‐TKI treatment was 39 m (19–83)	The median duration of uMR4.5 was 29 m (24–64)^e^	No	No	Not reported
Lee et al., 2013; Lee et al., 2016; Park et al., 2016; KID study	Lee et al., 2013; *n* = 48	58/42%	47 (median) (19–74)	85.3 m (median) (39.9–129.5)	UMRD^g^ 56.8 m (median) (24–105.7)	No	No	Not reported
	Lee et al., 2016; *n* = 90	58/42%	56.2 (median) (26–82)	80.8 m (median) (38.2–141.3)	UMRD^g^ 39.9 m (median) (22.1–130.7)	Yes	Yes	Aggravation or new development of musculoskeletal pain and/or pruritus after imatinib discontinuation was presented in 30% of patients
Ross et al., 2013; TWISTER	40	63/37%/43/57%^b^	58/62, median^b^	70/72 m^b^	UMRD 30/41 m (median)^b^	No	No	Not reported
Takahashi et al., 2012; multicentre retrospective study	43 out of 3242 imatinib treated patients	56/44%	Median age at dg: 57 years (18–80)	Imatinib treatment 45.2 m (median) (4.5–92.7)	The median duration of CMR before cessation was 27.4 m (0.9–79.6)^f^	No	No	Not reported

Abbreviations: AE = adverse events; dg = diagnosis; m = month; pts = patients; y = year.

^a^ musculoskeletal pain all grades during treatment‐free period (Mahon et al., [13]).

^b^ imatinib only/interferon‐imatinib.

^c^ MR ≤ 0.0069% = MR 4.2. Twenty‐one % of pts switched for resistance, 57% of pts switched for intolerance, and 22% of pts switched at the patient's request.

^d^ In total, 69% of pts who were off dasatinib treatment experienced any grade AEs (18%: grade 3/4). Any grade musculoskeletal and connective tissue AEs occurred in 39% of pts discontinuing dasatinib; arthralgia and arterial hypertension at any grade were the most common off‐treatment AEs, occurring in 18% and 13% of pts, respectively. A total of 15 withdrawal events were reported in nine patients (11%), all within 18 months of discontinuing treatment; the median (range) time from discontinuation to withdrawal event was 3.7 (0.3–17.9) months; withdrawal events were defined as any AE occurring and/or worsening due to treatment cessation, as determined by the investigator.

^e^ uMR4.5 = undetectable molecular transcript with the method with the sensitivity of MR4.5.

^f^ CMR = complete molecular response, not detectable *BCR::ABL1* transcript with a method with a sensitivity < 0.01%.

^g^ UMRD = undetectable molecular residual disease with the method with the sensitivity of at least MR4.5.

In the next text, the European LeukemiaNet (ELN) laboratory recommendations for the diagnosis and management of CML will be followed regarding the terminology of molecular responses. The MMR is defined as *BCR::ABL1* levels 0.1% on international scale (IS) and DMR (deep molecular response) as *BCR::ABL1* 0.01% and deeper [37]. Nevertheless, when appropriate, the original terminology of the authors of the discussed papers will be used too.

## 4 The Theoretical Background of Treatment Cessation in CML

CML is a disease of hematopoietic stem cells. Several years ago it had already been shown, that the Ph chromosome can be detected not only in all maturation stages of granulopoiesis, erythropoiesis, and megakaryocytes, but also in plasma cells, eosinophils, basophils and monocytes. Moreover, using immunological identification of single cells, the t(9;22) was detectable in CD3‐positive T lymphocytes, CD19‐positive B lymphocytes, and in CD34‐positive precursor cells [38]. A stem cell compartment hit is the reason why TKIs alone can rarely completely eradicate the malignant cells and cure the disease. It has been shown that *BCR::ABL1* is enriched in cells in the S and G_2_ phase, so in a cell phase dependent manner, preventing imatinib to be active in non‐cycling hematopoietic stem cells [39]. In *in vitro* experiments, CML stem cells remained viable in a quiescent state even in the presence of growth factors and imatinib [40]. Moreover, one report showed that human CML stem cells do not depend on *BCR::ABL1* activity to survive and are thus not eliminated by imatinib therapy; this finding suggests that primitive CML cells are not oncogene addicted and that therapies that biochemically target *BCR::ABL1* will not eliminate CML stem cells [41].

These experimental data are supported by clinical observations, and guidelines actually consider at least MMR, the *BCR::ABL1* transcript level ≤ 0.1%, not complete eradication of the disease, as an optimal long‐term response to TKIs [6], which is in fact still a molecular disease persisting with a reduction in starting disease burden by 3 orders of magnitude. Moreover, eliminating leukemic stem cells is not granted even after an allogeneic hematopoietic stem cell transplantation [42].

Based on data from imatinib treated patients, it has been shown that successful therapy leads to a biphasic exponential decline in leukemic cells. The first slope is determined by the decline between 0 and 3 months, with a mean value of 0.05 per day (5% decline per day) and represents the turnover rate of differentiated leukemic cells, while the second slope of 0.008 per day (0.8% per day) represents the turnover rate of leukemic progenitors between 6 and 12 months [43]. The average rate of the exponential increase after stopping treatment is 0.09 per day, corresponding to a doubling time of 8 days. The model suggests that imatinib is a potent inhibitor of differentiated leukemic cell production, but does not deplete leukemic stem cells [43]. Indeed, malignant cells’ persistence has been proven even in patients with undetectable *BCR::ABL1*; however, it has to be emphasized that the standard *BCR::ABL1* detection methods have sensitivity limitations. Chomel et al. [36] used purified CD34+ cells for clonogenic and long‐term culture‐initiating cell assays in patients with undetectable molecular residual disease and found *BCR::ABL1*‐expressing leukemic stem cells in all patients. Ross et al. [44] used highly sensitive patient‐specific nested quantitative PCR to look for evidence of genomic *BCR::ABL1* DNA in patients who sustained complete molecular response after stopping imatinib therapy. Seven out of eight of these patients had *BCR::ABL1* DNA detected at least once after stopping imatinib, but none has relapsed. *BCR::ABL1* DNA levels increased in all 10 patients who lost complete molecular response soon after imatinib cessation, whereas serial testing of patients in sustained complete molecular response showed a stable level of *BCR::ABL1* DNA. This more sensitive assay for *BCR::ABL1* provided evidence that even patients who maintain a complete molecular response after stopping imatinib may harbor residual leukemia. Pagani et al. [45] used the conventional method, RQ‐PCR for *BCR::ABL1* mRNA, reflecting a composite number of circulating leukemic cells and the *BCR::ABL1* transcripts per cell, and *BCR::ABL1* genomic DNA, reflecting only the leukemic cell number in parallel to determine the relative contribution of the leukemic cell number to molecular response. In the first 3 months of treatment, *BCR::ABL1* mRNA values declined more rapidly than DNA. By 6 months, the two measures aligned closely. *BCR::ABL1* DNA was also quantifiable in samples with undetectable *BCR::ABL1* mRNA. These parallel studies showed that the rapid decline in *BCR::ABL1* mRNA over the first 3 months of treatment is due to a reduction in both cell number and transcript level per cell, whereas beyond 3 months, falling *BCR:ABL1* mRNA levels are proportional to the depletion of leukemic cells.

Experimental and clinical data led to some CML TKI treatment models. Lenaerts et al. [46] hypothesized that TKI therapy can cure CML without hitting leukemic stem cells, although it may have to be prolonged. Stein et al. [47] postulated that the gradual decrease in *BCR::ABL1* levels seen in patients is due to a continual, gradual reduction in leukemic stem cells. Horn et al. [48] predicted that the majority of individuals require several decades of therapy before the disease is completely eradicated. The cumulative rates for complete eradication after 15 and 30 years of treatment are estimated to be 14% and 31%, respectively. In approximately 67% of the patients, residual leukemic cells are predicted throughout the remaining lifetime, assuming a life expectancy of 80 years. Furthermore, their model prognosticated that 31% of the patients will remain in deep molecular remission (MR5.0) after treatment cessation after a fixed period of 2 years in MR5.0, whereas 69% are expected to relapse.

Taken together, it is quite clear that the current TKI treatment, albeit being extraordinarily successful in disease control, can rarely completely eradicate the disease, and if possible, it would require long therapy. On the other hand, however, it is surprising and intriguing that treatment cessation is successful in around 50% of patients, and some patients can even be off therapy with persistent *BCR::ABL1* positivity. This might be explained by some intrinsic factors inside the leukemic stem cells, or by some extrinsic factors influencing the leukemic stem cells, either within the bone marrow niches, or by some immunological control mechanisms. Nevertheless, we have to admit these mechanisms are still largely unexplored and unknown.

## 5 Eligible Patients

There are two important aspects of eligibility for an attempt to discontinue CML treatment. The first concerns the disease status, the second the patients' wishes and expectations.

Generally, only patients in a chronic phase at diagnosis are considered for treatment discontinuation. A TKI switch for intolerance is allowed, but switching for resistance may be risky, since some trials which allowed resistant patients to enroll reported poorer outcome [25, 26, 29]. Stopping treatment might be cautiously considered in resistant patients with a mutation sensitive to another TKI [49]. Other important eligibility criteria are the duration of TKI therapy and the depth and/or duration of defined treatment response. Regarding these criteria, some trials were quite strict, while others quite flexible. See Table 1. Usually, at least 3 years of TKI therapy was required, however, the real duration was longer; for example, in the largest published study, EURO‐SKI, the median TKI duration was 7.5 years (3–14.1 years). The required response is usually at least MR4, but some trials required at least MR4.5, with a minimal duration of 1–2 years. Again, the real duration was longer than the required minimum; in the EURO‐SKI trial, median MR4 duration was 4.7 years (1–13.3 years). In order to precisely quantify *BCR::ABL1*, typical, or quantifiable transcript is required [37].

Despite imatinib, nilotinib and dasatinib discontinuation being widely tested, only nilotinib has an official label for treatment discontinuation in SPC (Summary of Product Characteristics) based on two trials organized by Novartis, ENESTop and ENESTfreedom [18, 19, 20, 21]. The ENESTop trial enrolled patients who had not achieved MR4.5 on imatinib and were then switched to nilotinib, ENESTfreedom enrolled patients treated with nilotinib upfront; both trials required achieving MR4.5. Detailed characteristics of these trials are in Tables 1 and 2.

In most trials listed in Table 1, there are no specified reasons to discontinue treatment. Some trials refer elective discontinuation, adverse events, diagnosis of second cancer, patient request due to cost, long undetectable residual disease, inconvenience due to daily doses, pregnancy/planning pregnancy, or patient preference [17, 30, 34]. In fact, not all patients are willing to stop the treatment for several reasons. We performed a survey based on an 18‐item questionnaire in all TFR eligible patients (*n* = 246), to whom participating in a discontinuations trial was proposed. In fact, 56 patients declined (22.8%) to participate. These patients had more frequently been having longer TKI treatment, on reduced doses of imatinib, females, had lower level of education, were elderly, retired, disabled or unemployed, and with a longer journey to a specialized hematology center. Surprisingly, the presence of TKI‐related adverse events was not among the factors influencing the decision to participate or not [50]. Taken together, the patients' point of view and attitude to ceasing treatment have to be seriously discussed and considered before any attempt at stopping.

## 6 Monitoring After Treatment Discontinuation

As is apparent from Table 1, the majority of relapses occur early after stopping treatment. According to an analysis published by Dulucq et al. [35], the probability of molecular recurrence in the time periods 0–6, 6–12, 12–18 and 18–24 months after the first stopping attempt was 35%, 8%, 3% and 3%, respectively. Therefore, in order to identify molecular recurrence, it is necessary to monitor patients frequently and have the result quickly. All studies apart from one listed in Table 1 reported the monitoring schedule exactly. Usually, after stopping, the monitoring interval is 4 weeks for the first 6–12 months. Some trials adopted 6–8 weeks intervals after the first 6 months of follow‐up. After 12 months, monitoring intervals are prolonged to 6–12 weeks, and after 24 months, monitoring is usually performed at 12 weeks intervals.

Shanmuganathan et al. [51] modeled the safe minimum frequency for molecular monitoring. They demonstrated that reduced monitoring frequency in the first 12 months of a TFR attempt is likely to be safe. Planned monitoring frequency of every 2 months in the first 6 months and every 3 months between 6 and 12 months may provide the best balance between reduced testing and minimizing delays in relapse detection and TKI recommencement. Patients and clinicians in settings without constraints on molecular monitoring may still prefer more frequent testing for the reassurance that it provides. They recommend re‐initiation of monthly *BCR::ABL1* testing at the loss of MR4.5, including in the few patients with fluctuating *BCR::ABL1* levels without MMR loss. *BCR::ABL1* can substantially increase in the time between detecting molecular relapse and restarting TKI therapy. This highlights the importance of a rapid turnaround time for *BCR::ABL1* results (and clinical action to the result), especially with reduced monitoring frequency. Less frequent monitoring would make TFR attempts more cost‐effective. More importantly, in some settings, reduced monitoring may enable clinicians to offer TFR to patients for whom the availability of molecular monitoring is a barrier. Nevertheless, further prospective studies are needed to validate this proposal prior to incorporating it into the current standard of care.

## 7 Trigger for Retreatment

When to restart the therapy, or how to define the molecular relapse or recurrence is a very important question. Different studies used different criteria, as specified in Table 1. Nevertheless, it seems practical to use MMR loss. This is based on the French study A‐STIM (According to STIM, Stop Imatinib study), which compared very strict criteria to define molecular relapse (criteria of STIM trial, see Table 1) and loss of MMR [52]. They analyzed 80 patients and the cumulative incidence of MMR loss was estimated as 35% at 12 months and 36% at 24 months. However, when using the strict criteria of STIM trial, cumulative CMR (complete molecular response) incidence loss was 51% at 12 months and 54% at 24 months. The explanation lies in the fact that some patients are weekly *BCR::ABL1* positive, yet below the MMR threshold, and this phenomenon is called the *BCR::ABL1* fluctuation.

## 8 Therapy Restart

Patients usually restart the same TKI which was discontinued. As is apparent from Table 1, the majority of patients usually quickly regain good molecular response. According to the analysis by Dulucq et al. [35], patients who resumed treatment re‐obtained DMR in 90% of cases with a median time of less than 6 months. Also, it is advisable to monitor patients more frequently after therapy reinitiation until a good molecular response is achieved. Molecular responses are usually evaluated every 4 weeks for the first 3–6 months and every 12 weeks thereafter, or as clinically indicated [15, 20, 21, 23, 24, 25, 26, 29, 30, 32].

## 9 Predictive Factors for Successful Therapy Cessation

Predictive factors for successful treatment cessation are very important, but unfortunately no strong, unified and generally accepted ones exist. Some potential predictive factors are easily available, others are more experimental and serve as a basis for further research. Easily available factors can be: age, gender, risk score, transcript type, duration of TKI therapy, duration of defined treatment response, depth of response, TKI used, and resistance to previous TKI therapy.

There are several outcome definitions regarding treatment cessation. Molecular recurrence‐free remission (MRFR) is measured as the time from TKI cessation to molecular recurrence, and TFR as the time from TKI cessation to molecular recurrence or TKI resumption (whichever comes first). MRFS (survival) is measured as the time from TKI cessation to molecular recurrence or death, and TFS (survival) as the time from TKI cessation to molecular recurrence, TKI resumption or death [53]. There is also a broader definition of TFS: time from the TFR start date to molecular recurrence, TKI reinitiation due to any cause, progression to accelerated phase or blast crisis, or death from any cause [20, 21].

### 9.1 Age

In one trial, older age ≥ 60 years was associated with better outcome [15], and another trial described the best outcome for patients ≥ 65 years, and the highest relapse rate for patients < 45 years [54]. Haddad et al. [17] found older age a positive prognostic marker in univariant, but not in multivariant analysis. On the other hand, Hughes et al. [18] defined an age of < 56 years as a good prognostic factor, whereas several trials found no correlation with age and outcome [13, 14, 17, 21, 22, 23, 24, 25, 26, 27, 28, 29, 30, 32, 33, 34, 55].

### 9.2 Gender

The influence of gender on the success of treatment discontinuation was analyzed in several trials with no clear effect detected [13, 14, 15, 18, 19, 21, 22, 23, 24, 25, 26, 27, 28, 29, 30, 32, 33, 34, 55, 56].

### 9.3 Risk Scores

The data are not uniform. Two trials found lower Sokal risk scores as favorable prognostic factors [20, 21, 27, 28], one trial found a high‐risk Sokal score as an adverse factor [56], other studies found no influence [13, 14, 15, 22, 23, 24, 25, 26, 29, 30, 32, 33, 34, 55], including other scoring systems, like EUTOS [13, 14, 23, 24, 27, 28], or Hasford (Euro) [13, 14, 23, 24], or ELST [23, 24].

### 9.4 Transcript Type

Two reports identified transcript e14a2 as favorable [13, 14, 57], whereas other trials found no correlation [30, 32, 56]. Data about patients with an atypical transcript are rare. Johnson‐Ansah et al. [58] published a series of 24 patients and the 12‐ and 60‐month probabilities of TFR in patients with sequences lacking exon a2 (e13a3 and e14a3) was 100%, while those in patients with exon a2 (e6a2, e8a2, e19a2) were 73.3% and 52.1%, respectively. Although TFR probabilities were markedly lower in the e19a2 subgroup than in the e6a2/e8a2 subgroup with 12‐month rates of 66.7% versus 83.3% and 60‐month rates of 25.8% versus 83.3%, the difference did not reach statistical significance.

### 9.5 Duration of TKI Therapy

Some trials found this factor important [13, 14, 15, 16, 20, 23, 24, 27, 28, 30, 32, 34], others not [17, 18, 19, 22, 25, 26, 29, 33, 55, 56]. In Theoretical background of treatment cessation in CML section, there are models described showing that with longer TKI treatment duration there might be a chance of leukemic stem cell eradication. There is no clear answer to the question what the optimal therapy duration is before the attempt to cease treatment, but there are some hints of practical importance. Nevertheless, this safe treatment duration may be different for different TKIs. For imatinib treated patients, the significant cutoffs are calculated as 4.3 years [34], 4.5 years [27, 28], 5.2 years [30], 6 years [59], or 6.2 years [60]. Saussele et al. [14] calculated a 5.8 years cutoff based on the EURO‐SKI trial data; the majority of patients received imatinib. DESTINY study estimated a non‐linear average decrease of around 8% in the proportion of recurrences per additional year on TKI in the trial overall [23, 24]; also in this trial, imatinib prevailed. Further data are in Table 1.

### 9.6 Duration of Defined Treatment Response and Depth of Response

These factors may be linked together; the longer the treatment duration, the higher the rate of deeper responses [61]. Several trials confirmed the relationship between longer duration of deep response and higher TFR rate [13, 14, 17, 18, 20, 34, 56], but not all [23, 24, 27, 28, 29, 30, 32]. As for the duration of TKI therapy, there is no clear answer what the optimal depth and duration of response before the attempt to cease treatment is, but there are some hints of practical importance. The interpretation of different trial results is complicated by their heterogeneity too. In EURO‐SKI trial (predominately imatinib treated patients), the cutoff regarding the duration of DMR for significant differences in relapse rates was 3.1 years. The observed proportion of patients without relapse was 32% and 66% for patients in DMR for 1–2 years versus > 9 years, respectively [14]. Kim et al. [59] calculated the cutoff for DMR duration in imatinib treated patients as 4.5 years. Haddad et al. [17] postulated the cutoff for DMR duration as 5 years. Laganà et al. [56] also found 5 years of stable DMR as the cutoff predictive for longer TFR. In the latter two studies, patients were treated with different TKIs. Other information concerning the significance of response duration are in Table 1.

In trials listed in the Table 1, a DMR was usually among the entry criteria. One exception is the DESTINY trial, which allowed patients in just at least MMR to be enrolled [23, 24]. The MRFS at 36 months was significantly lower for the MMR group than the DMR group, 36% versus. 72%, respectively. On the other hand, however, whether being in MR4 or deeper response makes any difference in TFR rate is less clear. In that DESTINY trial, being in MR4.5 at study conferred no advantage. Haddad et al. [17] separated patients into three different groups based on the depth and duration of response and compared their outcomes: patients with MR4.5 ≥ 5 years, patients with MR4 > 5 years but MR4.5 < 5 years, and patients with MR4 < 5 years. The 5‐year TFR rates were higher for those with longer deep molecular response durations: 87% for MR4.5 ≥ 5 years, 92% for MR4.5 < 5 years but MR4 ≥ 5 years, compared to 64% for those with MR4 < 5 years.

Laganà et al. [56] emphasize the importance of stable DMR as a positive predictive factor for TFR; a prior “unstable DMR” was defined as a previous molecular remission pattern characterized by at least one episode of DMR loss confirmed in two or more consecutive distinct molecular assessments performed with a minimum interval of 1 month to differentiate a true DMR loss from a transient or spurious fluctuation.

### 9.7 TKI Used

The type of TKI is probably less important [17, 23, 29, 55, 56] while the duration of therapy and duration and achievement of a certain response play a role.

### 9.8 Resistance to previous TKI

This is also a controversial issue. Some trials consider it a poor risk factor [25, 26, 29], others not [19, 56]. Claudiani et al. [49] reported 5 out of 10 patients who have successfully maintained a prolonged TFR (up to 4.7 years) despite a previous history of TKI failure and presence of *BCR::ABL1* kinase domain mutations, including T315I. All were in MR4 for at least 1 year. The authors conclude it is reasonable to speculate that when an effective alternative TKI is started promptly after mutation detection, achieving a deep molecular response overcomes the traditionally accepted adverse patient outcomes. TFR appears feasible in patients with previous mutations, however, a larger number of cases are required to determine the prognostic impact of mutations on TFR probability and the safety of this approach. Stopping TKI in patients with mutations outside clinical trials currently needs to be reserved for those patients with significant TKI‐related toxicity in the absence of alternative therapy and to be approached with caution.

### 9.9 Other Easily Obtainable Potential Prognostic Factors

These factors have usually only been tested in some trials and therefore, their power is limited. This includes presence of withdrawal syndrome [30], or previous allogeneic hematopoietic stem cell transplantation [32]. On the other hand, no correlation has been found for spleen size below the costal margin at diagnosis [13] or ECOC performance score [23]. The presence of additional chromosomal abnormalities does not seem to preclude successful TFR attempt [62, 63]. Lower peripheral blasts at diagnosis were found to be predictive in one report [13], but not in another [56].

There are several studies testing other predictive factors, which are not yet routine clinical practice. Nevertheless, they shed more light on TFR's complexity and serve as the basis for further research.

### 9.10 *BCR:ABL1* Kinetics

The speed of decrease in *BCR::ABL1* from the start of therapy is a marker of disease sensitivity. On the other hand, the speed of *BCR::ABL1* increase after ceasing treatment may characterize patients with a high risk of molecular recurrence. Shanmuganathan et al. [64] calculated the patient‐specific halving time of *BCR::ABL1* after commencing TKI therapy and found the rate of reduction in transcripts over the first 3 months of therapy a strong predictor of sustained TFR. Nevertheless, this finding has not been confirmed by Saugues et al. [65]. They showed that early transcript kinetics was a predictor for optimal response, but not for TFR success. Kim et al. [66] calculated the so‐called *BCR::ABL1* doubling time upon TKI cessation. Doubling time is the number of days required for *BCR::ABL1* to double the previous expression level, and this parameter was calculated based on monthly‐monitored transcript values. The optimal doubling time value was determined as 12.75 days at 2 months. The patients were stratified into three groups: a high‐risk group (doubling time < 12.75 days but > 0, with rapidly proliferating CML cells) showed the lowest MRFS of 7.7% at 12 months, compared to 53.6% in the intermediate‐risk group (doubling time ≥ 12.75 days, with slowly proliferating CML cells) or 90.0% in the low‐risk group (doubling time ≤ 0, i.e., without proliferating CML cells). Monthly assessment of doubling time can help to identify high‐risk patients for treatment‐free remission failure with an imminent risk of molecular recurrence, and to define low‐risk patients who can be spared the frequent monitoring of monthly molecular tests. Analogous analysis was published by Gottschalk et al. [67] based on the data from the DESTINY trial; the trial design comprised initial de‐escalation to half the standard TKI dose for 12 months, followed by complete cessation. The authors conclude that the individual molecular dynamics during TKI dose reduction is a promising predictor for a molecular recurrence after TKI cessation. In particular, the exclusion of patients with steeply increasing *BCR::ABL1* slopes during the dose reduction period from complete TKI cessation is likely to reduce the overall number of recurrences and increase the TFR success rate on stopping TKI.

### 9.11 Special Methods for *BCR::ABL1* Detection

Pagani et al. [68] prospectively measured *BCR::ABL1* DNA as a predictive yes/no binary test in 5 cellular fractions from patients meeting conventional criteria to discontinue TKI. The median *BCR::ABL1* DNA level was higher in granulocytes and T cells, but not in other lineages, in patients who relapsed. Among patients undergoing their first TFR attempt, they defined 3 groups with differing relapse risk: granulocyte‐positive group (100%), granulocyte‐negative/T‐cell–positive group (67%), and granulocyte‐negative/T‐cell–negative group (25%). Machová Poláková et al. [69] compared DNA and mRNA *BCR::ABL1* measurements by qPCR and found, that patients who were DNA negative/RNA negative had the highest MRFS rate, followed by those who were DNA positive/RNA negative, and the DNA positive/RNA positive group had the lowest MRFS rate.

Several papers reported advantages in digital (or digital droplet) PCR for *BCR::ABL1* measurements [54, 55, 70, 71]; this is a relatively new quantification technique with improved precision and sensitivity compared to conventional real‐time quantitative PCR. In digital PCR, the sample is partitioned in thousands of micro‐particles prior to PCR, which enables precise and absolute *BCR::ABL1* transcript quantification even in low molecular disease levels. Lu et al. [70] reported median sensitivity in digital PCR 5.2 logs, and MR5 was achieved in the majority of samples; digital PCR gave positive results also in patients negative by conventional real‐time quantitative PCR. Mori et al. [54] achieved a digital PCR sensitivity as low as 10^−7^. Atallah et al. [55] examined all samples with an undetectable *BCR::ABL1* (< MR4.5) using droplet digital PCR, which offers approximately 0.5–1 log greater sensitivity. Molecular recurrence for patients with detectable *BCR::ABL1* transcripts at the time of study enrollment by standard PCR, undetectable transcripts by standard PCR but detectable by digital PCR, and for patients with undetectable transcripts by both techniques was 50%, 64.3%, and 10.3%, respectively (*p* ≤ 0.001). Two works showed cut‐off 0.0023% by digital PCR as predictive for successful TFR [60, 71]. This level corresponds to MR4.64 and it is quite intriguing that such relatively small differences in minimal residual disease should have such a huge effect on TFR. At the time of CML diagnosis, the number of malignant cells is approximately 10^12^ [72], which is a mass of approximately 1 kg. MR4 means a reduction by 4 logs, so to the level of about 10^8^ cells, which is still a significant number of cells, albeit of minimal weight (about 0.1 g), and MR5 means still at least 10^7^ residual malignant cells.

### 9.12 Other Molecular Markers

Machová Poláková et al. [73] described single nucleotide polymorphism rs460089 located in the promotor of *SLC22A4* gene encoding imatinib transporter OCTN1 as an independent predictor of TFR. Another work tested the effect of ABCG2, OCT1, and ABCB1 (MDR1) transporters' gene expressions and found that the lower transcript levels of the efflux transporter ABCG2 predicted TFR after TKI discontinuation [74]. Extensive proteomic profiling of 162 different plasma proteins, however, could not predict TFR [75].

## 10 Immunological Aspects of TFR

Since several patients keep their TFR despite having slightly positive or fluctuating levels of *BCR::ABL1* transcript, there must be some mechanisms keeping the leukemic cells in a non‐proliferating status. From the era of allogeneic hematopoietic stem cell transplantation for CML we know that immune mechanisms actually exert extremely potent antileukemic effect [76, 77]. Therefore, one intensively studied aspect of TFR is immune mediated residual leukemic cell control. If elucidated, we would be able to harness these putative immune mechanisms to keep patients off therapy. However, despite several works dealing with this topic, our knowledge is still far from being complete. No predictive marker is in routine clinical use, nor any immunological intervention to potentiate anti‐leukemic effect. A brief summary of these studies is in Table 3. From this Table, it is apparent that no single simple immunological marker correlated with TFR maintenance. Nevertheless, it seems that some active NK cell subsets play an important role. Moreover, patient cells holding TFR have a less exhaustive or suppressive character. Lastly, T cells in these patients are enriched by those actively targeting the leukemic cells.

**TABLE 3:** Summary of key papers dealing with immunological aspects of TFR.

Author Publication year	Key findings
Decroos et al. 2024 [78]	Perforin, a key cytotoxic immune player, was expressed in a significantly higher proportion of both innate CD8 T‐cell and NK‐cell subsets in non‐relapsed pts, compared with relapsed pts at D0. For all T‐cell subsets, surface‐expression level of PD‐1, an exhaustion marker, decreased in non‐relapsed patients compared with relapsed patients at D0. Lastly, a negative correlation between the proportion of innate CD8 T‐cells expressing PD‐1 and those expressing perforin in non‐relapsed patients at D0 was found.
Huuhtanen et al., 2024 [79]	With the in‐depth analysis of cellular and molecular immune responses in CML, including profiling of T cells specific to leukemia‐associated antigen PR1 with TCRβ‐sequencing, the authors identified the active NK cells and anti‐PR1 T cells that could help maintain TFR in pts discontinuing TKI treatment
Kwaśnik et al., 2024 [80]	Significant increase in the percentage of CD8^+^PD‐1^+^ cells in patients losing TFR. The level of CD8^+^PD‐1^+^ cells was inversely related to the duration of treatment and incidence of deep molecular response before discontinuation
Ureshino et al., 2024 [81]	The combination of *KIR3DL1*‐*HLA‐Bw4* (an *HLA‐B* allele) was an independent factor for a higher risk of molecular relapse. Pts at higher risk of relapse also had a significantly lower number of NK cells at TKI discontinuation than the other patients (CD16^+^/CD56^+^ NK cells)
Irani et al., 2023 [82]	Absolute numbers of T‐regs were increased in pts who experienced molecular relapse (MolR) compared with those who sustained TFR. The immune‐checkpoint receptors PD‐1, CTLA‐4, LAG‐3, and TIGIT on T or NK cells were not differentially expressed between the MolR and TFR groups. However, TIM‐3 was consistently upregulated on bulk T cells (CD3^+^) and T‐cell subsets including, CD4^+^ T cells, CD8^+^ T cells, and T‐regs, in pts who relapsed in comparison with those who maintained TFR after discontinuation. TIM‐3 is involved in T cell exhaustion and other immune cell functions
Irani et al., 2020 [83]	NK cells with increased expression of activating NK receptors were higher in pts who achieved TFR. There was no difference in the proportion of CD4+ or CD8+ T cells. Furthermore, FoxP3+ regulatory T cells and monocytic myeloid‐derived suppressor cells were concomitantly decreased in TFR pts, suggesting that the effector and suppressor arms of the immune system work in concert to mediate TFR.
Austin et al., 2019 [84]	Analysis of the DESTINY trial (see Table 1). No significant association was seen between any lymphocyte subset changes in the first 6 months and molecular relapse. However, a rise in effector memory CD8^+^ cells between 6 and 12 months was associated with an increased risk of molecular recurrence; a similar effect was seen for this subset between baseline and 12 months. No association was seen between the 6 to 12‐ or 0–12‐month trends in any other subset and molecular recurrence.
Cayssials et al. 2019 [85]	Dramatic increase of functionally active innate CD8(+) T‐cells in patients in TFR ≥ 2 years as compared to control subjects and patients in remission under tyrosine kinase inhibitors
Okada et al., 2018 [25]	The univariate analysis revealed that a greater number of total NK cells (CD3^−^CD56^+^) and cytolytic NK cells (CD16^+^CD56^+^) and a lower number of γδ^+^ T cells and CD4^+^ Tregs were associated with successful maintenance of TFR. Multivariate analysis revealed that only the greater number of cytolytic NK cells (CD16^+^CD56^+^) were independently associated with maintenance of TFR. When the number of total NK cells (CD3^−^CD56^+^) rather than the number of cytolytic NK cells was entered into multivariate analysis, the number of total NK cells was no longer a significant factor
Ilander et al., 2017 [86]	The proportion of NK cells was associated with the molecular relapse‐free survival as pts with higher than median NK‐cell percentage at the time of drug discontinuation had better probability to stay in remission. Similar association was not found with T or B cells or their subsets. In non‐relapsing pts the NK‐cell phenotype was mature, whereas pts with more naïve CD56^bright^ NK cells had decreased relapse‐free survival. In addition, the TNF‐α/IFN‐γ cytokine secretion by NK cells correlated with the successful drug discontinuation.
Rea et al., 2017 [87]	At the time of imatinib discontinuation, non‐relapsing pts had significantly higher numbers of NK cells of the cytotoxic CD56^dim^ subset than had relapsing pts, while CD56^bright^ NK cells, T cells and their subsets did not differ significantly. After imatinib cessation, the NK cell count increased significantly and stayed higher in non‐relapsing pts than in relapsing patients, while receptor expression and functional properties remained unchanged.
Schütz et al., 2017 [88]	In a prospective analysis of pts discontinuing their TKI, the 1‐year relapse‐free survival was 30.1% for patients with > 95 CD86^+^pDC per 10^5^ lymphocytes (plasmacytoid dendritic cells), but 70.0% for patients with < 95 CD86^+^pDC. High CD86^+^pDC counts significantly correlated with leukemia‐specific CD8^+^ T‐cell exhaustion. CD86 is the T‐cell inhibitory receptor (CTLA‐4) ligand
Caocci et al., 2015 [89]	Cumulative TFR was significantly higher in pts homozygous for KIR A haplotype. Bx haplotype, and the combination KIR3DS1/KIR3DL1 present/HLA‐Bw4 present were significantly associated with relapse

Abbreviations: NK = natural killer, pts = patients, y = year.

## 11 Potential Complications of TKI Withdrawal

### 11.1 Withdrawal Syndrome

Richter et al. [90] referred a substantial rate of patients for the first time in 2014 who, somewhat unexpectedly, reported musculoskeletal pain that had begun or worsened within weeks after stopping imatinib. The authors called these symptoms withdrawal syndrome (WS). Since that publication, more attention has been paid to this problem and we now know that the frequency of WS is around 20%–40% and that WS is a TKI class effect, see Table 1 Risk factors for developing WS might be low body weight and body mass index, longer duration of TKI treatment with a cut‐off time of 93 months, and history of osteoarticular symptoms [91, 92]. Another study reported a higher frequency in females, but no difference regarding the length of TKI therapy (cut‐off 43.5 months) and no clear difference regarding the history of musculoskeletal troubles [21]. WS starts within 1–12 weeks after TKI discontinuation and manifests as pain localized to various part of the body, including the shoulder and hip regions, extremities, and/or hands/fees, fingers and/or wrists, cervical spine, or as muscle tenderness, arthralgia, myalgia, muscle cramps, bone pain, whereas at other times it may resemble polymyalgia rheumatica [23, 24, 90, 91, 92]. In the ENESTfreedom study [21], the majority of patients had grade 1/2 side effects. Laboratory evaluation does not bring any significant information, because electrolytes, creatine kinase, C‐reactive protein, and serum protein electrophoresis are normal, or marginally increased [90, 91]. Other side effects are reported upon treatment cessation, too, like hypertension [15], or pruritus [30].

The pathogenesis of WS is not clear. It has been shown that TKIs actually bind many different targets inside the cells with comprehensive drug‐protein interaction profiling by chemical proteomics [93]; so, we might hypothesize, that after many years of TKI therapy the organism adapts to this situation, and abruptly stopping treatment creates new disturbances. WS therapy is therefore not causative, and paracetamol, non‐steroidal anti‐inflammatory drugs, a short course of steroids, or TKI restart can be considered and used [90, 92]. WS usually lasts 3–30 months, with median duration of 7 months [91, 92].

### 11.2 Late Molecular Relapses

As is apparent from Table 1 and from the analysis of Dulucq et al. [35] (see also the paragraph Monitoring after treatment discontinuation), the vast majority of molecular recurrences after abruptly stopping occur in the first 6–12 months. Nevertheless, late relapses do exist too, and in clinical practices we must be aware of this fact. Several works have dealt with this important aspect of treatment cessation and it is clear that a long‐term molecular follow‐up remains mandatory for CML patients in TFR.

Rousselot et al. [94] reported 18% late molecular recurrences after 2 years in TFR and they occurred in patients with fluctuating MRD measurements. Richter et al. [95] collected the long‐term data for the EURO‐SKI trial and found three different *BCR::ABL1* levels patterns beyond 36 months after stopping: continuously undetectable *BCR::ABL1*, occasionally detectable values, or fluctuating levels in 54%, 20%, and 26% of patients, respectively. With a 72‐month follow‐up, 12 out of 111 patients (10.8%) who were in TFR at 36 months, subsequently lost MMR. These relapses occurred between months 40 and 72 (median 51 months) after stopping TKI. The *BCR::ABL1* level at relapse was between 0.1% and 0.2% in all of these patients. The relapse kinetics was slow with an ∼1.5 fold rise in *BCR::ABL1* per month prior to losing MMR, which differed from the rapid relapses seen during the first 6 months after stopping TKI. All patients who restarted TKI therapy regained MMR within 1–5 months (median 3 months) after restarting. All patients except one also regained MR4 within 1–5 months (median 3 months) after restarting TKI. Their status at 36 months appeared highly predictive of a later relapse as only 1 patient out of 98 in MR4 at month 36 lost MMR in the following 3 years. Conversely, 11 of the 13 patients not in MR4 at month 36 lost MMR during follow‐up. Dulucq et al. [53] emphasized the different patterns of late versus early molecular relapses. With a median follow‐up of 40.8 months (5.5–111 months), the probability of TFR was 43.4% at 5 years, 40.9% at 7 years, and 34.5% at 9 years. Molecular recurrence occurred between 0 and 6 months, 6–24 months and after 24 months in 69%, 14% and 17% of patients, respectively. Notably, the molecular recurrence kinetics differed significantly between these three subgroups with a median time from loss of MR4 to loss of MMR of 1, 7 and 22 months, respectively. Predictive factors for molecular recurrence differed, and those factors predicting early recurrences were not significant for later ones. Despite the kinetics of late molecular recurrences seeming to be slow, there are exceptions. Katagiri et al. [96] referred to patient being in TFR for more than 7 years but then relapsing with relatively rapid *BCR::ABL1* elevation.

Ross et a [97]. published an intriguing observation illustrating our so far superficial understanding of processes keeping the leukemic stem cells dormant upon treatment discontinuation. Nine patients in long‐term TFR were monitored by highly sensitive individualized *BCR::ABL1* DNA PCR in a sufficient number of samples to enable more precise residual leukemia quantification. *BCR::ABL1* DNA decreased from a median of MR5.0 in the first year of TFR to MR6.1 in the sixth year of TFR. This serial high sensitivity testing provided a new and unexpected finding of gradually reducing CML cells in some patients in long‐term TFR.

### 11.3 Blast Crisis Development

Blast crisis (BC) is a dreaded catastrophic development in the CML course. There are a couple of reports of BC emergence in patients being in TFR, or shortly after TKI resumption for molecular recurrence [52, 98, 99, 100, 101, 102, 103]. Three patients developed lymphoid BC shortly after TKI retreatment for molecular relapses, however, one patient had poor compliance, and all patients are alive. Eleven patients developed BC when off therapy, however, one patient self‐discontinued treatment in the setting of poor disease control, and another patient discontinued treatment because of plans to have a child; also in this patient, the disease was not ideally controlled. Out of these 11 patients, 9 had a lymphoid BC, whereas 2 developed a myeloid BC, and 9 patients were alive at the time of the above‐mentioned publications. BC appeared after 6–63 months following treatment cessation, and BC emergence is usually rapid a few months from the last MMR, sometimes sudden without a preceding significant *BCR::ABL1* increase. Some patients had additional chromosomal or genetic abnormalities, or *BCR::ABL1* mutations. Several treatment options were employed, like TKIs, chemotherapy, blinatumomab, or hematopoietic stem cell transplantation. Nevertheless, the probability of BC in real life is presumably very low. Zambrotta et al. [98] analyzed 870 patients eligible for treatment discontinuation or who stopped the treatment and concluded that the low likelihood of disease progression and the lack of an evident relationship between disease progression and treatment discontinuation represent useful and encouraging information that should allay fears of disease progression among patients who attempt to cease treatment.

## 12 Kinetics of Preexisting TKI‐Related Side Effects, Laboratory Values, and Quality of Life Upon Treatment Cessation

This is in fact a very important point, since TKIs can have plenty of very different side effects, including serious ones, as can be seen from the current SPCs of particular TKIs, or from extensive reviews [104, 105]. Actually, and surprisingly, despite considering these aspects of ceasing TKI as fundamental, out of 14 trials listed in the Table 2, only 4 dealt with the kinetics of preexisting TKI‐related side effects [18, 19, 20, 21, 23, 24, 31], and 3 with laboratory value kinetics [18, 19, 20, 21, 31].

### 12.1 Kinetics of Preexisting TKI‐Related Side Effects

One could intuitively assume, that these side effects diminish; however, the reality is much more complex, and the surprising new phenomenon, withdrawal syndrome, can also occur in a significant proportion of patients. Anyway, alleviation has its kinetics and not all patients reported all symptoms disappearing. Different trials captured different side effects and also differed in methodologies, so some results are not concordant. In the DESTINY trial [23, 24], many patients described symptoms present at trial entry, either in verbal reporting at scheduled visits or in their diaries. Individual side‐effects (lethargy, diarrhea, rash, nausea, periorbital edema, and hair thinning) all improved in the first 3 months of de‐escalation, but not thereafter. However, little further improvement was observed in the subsequent 9 months. In the LAST trial [55], fatigue, depression, diarrhea, sleep disturbance, and pain interference were studied. Fatigue improved after TKI discontinuation, and the improvement was sustained over time (25.6% and 80.4% of patients had a clinically meaningful improvement at 6 and 12 months respectively). Improvement in depression, diarrhea, and sleep disturbance was reported in 34.8%, 87.7%, and 21.4% of patients, respectively, at 12 months of TFR. The mean pain interference score worsened slightly after discontinuing TKI, but the change was not statistically or clinically significant. Very few patients reported a clinically meaningful worsening in pain interference score, whereas some reported a clinically meaningful improvement at 12 months. Park et al. [31] studied several parameters at 6 months after stopping TKI and 3 patterns were registered: improvement, no change, and worsening. For the following parameters, improvement prevailed over worsening: easy bruising, easy bleeding, cold intolerance, night sweat, chilling, night spasms, edema, hair color turning white, skin fragility, skin color turns white, abdominal discomfort, indigestion, vomiting, nausea, and anorexia. EURO‐SKI trial also provided valuable information regarding the kinetics of some preexisting side effects upon treatment cessation [106]. In all age groups, statistically significant improvements were observed with regard to nausea/vomiting and diarrhea. With regard to fatigue, statistically significant improvements were only observed for the younger groups: 18–39 years (*p* < 0.001) and 40–59 years (*p* = 0.003). For the eldest group (≥ 70 years), a statistically significant deterioration over time was found (*p* = 0.039). A significant worsening of pain over time was observed within all age groups except for those aged between 18 and 39 years. Twenty‐three percent of patients reported a sustained clinically meaningful increase in pain during the 12 months after stopping TKIs. In the multivariate model, the probability of experiencing worse pain was independently associated with older age at the time of stopping TKIs and with a longer duration of TKI treatment. No significant differences over time were observed in any of the age groups with regard to insomnia, appetite loss, and constipation.

ENEST trials [18, 19, 20, 21] provide short‐term as well as long‐term follow‐up data. In the ENESTfreedom study, the frequency of side effects during the 1‐year nilotinib consolidation phase versus 1‐year TFR phase were as follows: nasopharyngitis 11.1% versus 8.4%, arthralgia 8.4% versus 12.1%, hypertension 7.9% versus 3.7%, diarrhea 5.8% versus 4.2%, headache 5.2% versus 5.3%, pain in an extremity 2.6% versus 6.3%, musculoskeletal pain 16.3% versus 24.7%, fluid retention 2.1% versus 4.2%, rash 4.2% versus 1.1%, and pancreatitis 1.6% versus 0%. In the long‐term follow‐up, the frequency of different side effects gradually decreased to 10.5% in the 5^th^ year of TFR, from 31.4% in the consolidation year and 44.7% in the 1^st^ year of TFR. There were 4.7% of cardiovascular events during the consolidation phase and 2.3% of these side effects in the 5^th^ year of TFR. In the ENESTop study, the frequency of side effects during the 1‐year nilotinib consolidation phase versus 1‐year TFR phase were as follows: nasopharyngitis 6% versus 4%, arthralgia 6% versus 23%, hypertension 7% versus 6%, pain in extremity 6% versus 7%, myalgia 3% versus 13%, musculoskeletal pain in general 14% versus 42%, fluid retention 2% versus 10%, and rash 5% versus 0%. In the long‐term follow‐up, the frequency of different side effects gradually decreased to 22.8% in the 5^th^ year of TFR, from the 22.8% in the consolidation year and 61.4% in the 1^st^ year of TFR. There were 1.8% of cardiovascular events during the consolidation phase and the same frequency of these side effects in the 5^th^ year of TFR.

An important aspect of the preexisting TKI‐related side effects is their frequency upon treatment re‐initiation. In the ENESTfreedom study, the most frequent all‐grade adverse effects of special interest reported during the treatment re‐initiation phase were musculoskeletal pain (19.8%), increase in blood cholesterol (18.7%), and cardiovascular events (17.6%). Overall, there was a considerable increase in all‐grade clinically notable adverse events in the treatment re‐initiation phase compared with the consolidation phase [20]. In the ENESTop trial, the rate of all‐grade adverse effects in patients who re‐initiated nilotinib was higher than during TFR; the most frequent adverse effects were hypertension, (28.8%), constipation, arthralgia, and hyperglycemia (all 15.3%). Overall, there was a considerable increase in all‐grade clinically notable adverse effects in the treatment reinitiation phase compared with the consolidation phase, including a higher frequency of cardiovascular events [18].

### 12.2 Kinetics of Laboratory Values

These data from 3 trials are summarized in Table 4. Briefly, blood count tends to improve, as well as other laboratory parameters, like ALT, AST, bilirubin, phosphate, creatinine, calcium, lipase, or ALP. Curiously, Park et al. [31] reported a very significant increase in cholesterol levels after imatinib cessation. We also noticed this phenomenon, together with decreased adiponectin levels [107]. The explanation probably lies in the fact that imatinib significantly increases adiponectin levels, together with decreasing cholesterol levels, as we previously showed [108]; so, after imatinib discontinuation, the level of adiponectin decreases which corresponds with the increase in lipid levels.

**TABLE 4:** Kinetics of laboratory values upon treatment stopping. Toxicities of any grade are listed.

Laboratory parameter before stopping/in TFR	Trial; number of patients; drug		
	Hughes et al., 2021 [18]; Mahon et al., 2018 [19]; ENESTop; *n* = 126; nilotinib	Radich 2021 [20]; Hochhaus 2017 [21]; ENESTfreedom; *n* = 190; nilotinib	Park et al., 2016 [31]; Kid study; *N* = 55; imatinib
Anemia	29%/18%	24.2%/20%	Significant increase in hemoglobin
Lymphopenia	17%/10%	8.9%/13.7%	
Thrombocytopenia	14%/13%	8.4%/10.5%	Significant increase in platelets
Leukopenia	7%/10%	2.6%/5.8%	Significant increase in leukocytes
Increased ALT	52%/15%	37.4%/12.6%	—
Increased AST	44%/11%	15.8%/6.8%	—
Increased bilirubin	33%/4%	30.0%/3.2%	—
Decreased phosphate	33%/5%	—	—
Increased creatinine	31%/26%	—	Decrease in creatinine
Decreased calcium	23%/4%	—	—
Abnormal uric acid	22%/32%	—	—
Increased lipase	20%/10%	30.0%/11.6%	—
Increased ALP	18%/10%	—	—
Increased cholesterol	0%/0%‐3.5%^a^	3.5%/0%–4.7%^a^	Significant increase
Increased glucose	1.8%/0%–5.3%^a^	39.5%/19.5%	—
Albumin	—	—	Significant increase

^a^ during 5 years follow‐up.

### 12.3 Quality of Life (QoL)

Surprisingly, no dramatic changes upon stopping treatment are evident. One reason might be that the instruments used so far for measuring the toxic effects and QoL are not optimal [109]. In the ENESTfreedom study, minimal changes in QoL scores after stopping or reinitiating treatment were detected among evaluable patients [21]. The proportions of patients reporting problems in each dimension of the EQ‐5D‐5 L tended to be similar across study phases, although a slightly higher proportion of patients reported problems (of any severity) with pain/discomfort after stopping treatment. In the DESTINY trial [23, 24], functional assessment of cancer therapy‐biologic response modifiers (FACT‐BRM) and EuroQol 5 dimensions QoL data at trial entry were already similar to a healthy control population, suggesting that these instruments are too insensitive for well controlled chronic myeloid leukemia. Formal QoL assessments were of marginal use and did not change during de‐escalation. An analysis of QoL in the EURO‐SKI trial [106] indicated that QoL and symptom trajectories may vary depending on specific age groups, with younger patients benefiting the most. Improvements in patients aged 60 years or older were marginal across several QoL and symptom domains.

## 13 Conclusion

After early attempts to stop interferon, even before the TKI era, rigorous testing to discontinue imatinib started some years after the advent of this new CML therapy. Since that time, an enormous amount of knowledge has been gathered, however, despite many trials and studies in this field, there are still significant gaps, and many fundamental questions remain unanswered. CML is a hematopoietic stem cell disorder; therefore, TKIs cannot completely eliminate the dormant stem cells and usually only lead to deep disease suppression. So, probably the most intriguing question is the persistence of minimal residual disease, which, rather surprisingly, does not lead to disease recurrence in all patients. Immunity likely plays a strong role, but the detailed aspects remain to be elucidated. Nevertheless, today's understanding enables safely discontinuing TKI in eligible patients outside clinical trials. Notwithstanding, TKI cessation still has to be considered and indicated with caution, taking into account several important aspects. Actually, the first crucial aspect is why to stop the treatment at all? There can be some long‐term treatment side effects, it might be the patient's wish, planned pregnancy, or perhaps some financial issues due to drug prices. But there are other questions, like: (i) are all the eligible patients willing to cease the treatment? (ii) will all the TKI‐related side effects relieve upon stopping? (iii) are there any side effects after discontinuing treatment?

Based on available data, we can summarize that: (1) the trials usually included patients in chronic phase with no resistance to previous TKI therapy, and with no age limit; (2) minimum TKI pre‐treatment was typically around 3 years; (3) achieving some defined treatment responses was required, mainly MR4‐MR4.5; (4) as a trigger for re‐treatment, generally losing MMR was selected; (5) after abruptly stopping treatment, the majority of molecular relapses occurred in the first 6–12 months, but late relapses did occur, too; (6) the long‐term TFR rate is around 50%; (7) after stopping treatment, more frequent *BCR::ABL1* monitoring is employed; (8) among prognostic factors for successful TFR, the duration of previous TKI therapy and/or defined molecular response may play a role; (9) upon re‐treatment for molecular relapse, regaining deep molecular response is typically quick.

Today, we know, that not all potential candidates for treatment cessation desire to do that for several different reasons. Many routine as well as very sophisticated predictive factors have been tested, however, with contradictory results, and none are 100% reliable. Rather surprisingly, the preexisting TKI‐related side effects are not all immediately alleviated upon treatment discontinuation. For some it takes longer time, and some seem to be influenced a little. Moreover, a new phenomenon has been discovered, withdrawal syndrome, appearing in up to about 40% of patients after discontinuing treatment, consisting of musculoskeletal pain. Last but not least, there are late relapses, so the patients have to be monitored closely indefinitely.

Achieving TFR is now one of the goals of the CML therapy [6, 110]. Dasatinib, nilotinib, bosutinib, as well as asciminib, when used in the front‐line setting, produce deeper and faster molecular responses than imatinib [111, 112, 113, 114, 115], despite the overall survival advantage not being demonstrated [110]. Therefore, one of these drugs may be considered the first line treatment in newly diagnosed patients, if attempting TFR is the potential aim, taking into account the side effects and contraindications. This approach is now being tested in the SUSTRENIM randomized trial (International, Prospective Study Comparing Nilotinib vs. Imatinib with Early Switch to Nilotinib to Obtain Sustained Treatment‐Free Remission in Patients with Chronic Myeloid Leukemia) [116, 117]. Whether the newer generation TKIs like asciminib or a combined treatment strategy as frontline therapy will increase the rate of durable DMR/TFR or shorten the durable DMR to < 5 years to achieve a high TFR rate is possible but is an open research question [110, 116].

So, the art of treatment cessation is to select the best candidate based on many diverse factors and information, and to follow the patient in the most rational way with smart anticipation of potential risks and side effects.

## Funding

The author received no specific funding for this work.

## Conflicts of Interest

The author declares no conflicts of interest.

## Data Availability

Data sharing not applicable to this article as no datasets were generated or analysed during the current study.
